# Analyzing Spanish-Language Public Sentiment in the Context of a Pandemic and Social Unrest: The Panama Case

**DOI:** 10.3390/ijerph191610328

**Published:** 2022-08-19

**Authors:** Fernando Arias, Ariel Guerra-Adames, Maytee Zambrano, Efraín Quintero-Guerra, Nathalia Tejedor-Flores

**Affiliations:** 1 Research Group on Advanced Technologies of Telecommunications and Signal Processing (GITTS), Faculty of Electrical Engineering, Technological University of Panama, Panama City 0819-07289, Panama; 2 Centro de Estudios Multidisciplinarios en Ciencias, Ingeniería y Tecnología AIP (CEMCIT-AIP), Technological University of Panama, Panama City 0819-07289, Panama; 3 Centro de Investigación e Innovación Eléctrica, Mecánica y de la Industria (CINEMI), Technological University of Panama, Panama City 0819-07289, Panama; 4 Centro de Investigaciones Hidráulicas e Hidrotécnicas (CIHH), Technological University of Panama, Panama City 0819-07289, Panama

**Keywords:** sentiment analysis, COVID-19, public health, social media, machine learning, natural language processing

## Abstract

Over the past decade, an increase in global connectivity and social media users has changed the way in which opinions and sentiments are shared. Platforms such as Twitter can act as public forums for expressing opinions on non-personal matters, but often also as an outlet for individuals to share their feelings and personal thoughts. This becomes especially evident during times of crisis, such as a massive civil disorder or a pandemic. This study proposes the estimation and analysis of sentiments expressed by Twitter users of the Republic of Panama during the years 2019 and 2020. The proposed workflow is comprised of the extraction, quantification, processing and analysis of Spanish-language Twitter data based on Sentiment Analysis. This case of study highlights the importance of developing natural language processing resources explicitly devised for supporting opinion mining applications in Latin American countries, where language regionalisms can drastically change the lexicon on each country. A comparative analysis performed between popular machine learning algorithms demonstrated that a version of a distributed gradient boosting algorithm could infer sentiment polarity contained in Spanish text in an accurate and time-effective manner. This algorithm is the tool used to analyze over 20 million tweets produced between the years of 2019 and 2020 by residents of the Republic of Panama, accurately displaying strong sentiment responses to events occurred in the country over the two years that the analysis performed spanned. The obtained results highlight the potential that methodologies such as the one proposed in this study could have for transparent government monitoring of responses to public policies on a population scale.

## 1. Introduction

As global societies find themselves increasingly connected to the internet, social networks have kept gaining relevance as forums where individuals not only share their opinions on events in their surroundings, but also publicly express their feelings or personal thoughts on a frequent basis. This is evident in virtually every country with access to any form of social media platforms, as a growing number of studies using data sourced from these sources continue to prove the relationship between real events and the reflected public sentiment observed on social media [1,2,3]. As a product of the ongoing pandemic, social media has become an essential tool for governments and companies alike to constantly provide guidelines and information related to the latest measures for containing the spread of the SARS-CoV-2 virus while attempting to keep economies afloat. This was especially true during the early phases of the pandemic, when little was known about the virus and containment measures were strict, resulting in a dramatic increase of opinions shared on social media by individuals [4].

The analysis of social media data predates the past three years due to pandemic stage in the world, and also related to various other health problems such as Heart Attack, Depression, Diabetes, Cancer and Tuberculosis [5]. In mental health, using sentimental analysis tools, Ref. [6] developed an Empathy-Centric Counseling Chatbot System, capable of sentimental dialogue analysis with students. Also, Ref. [7] presented a recent study about detecting sentiment dynamics and clusters of Twitter users for trending topics in COVID-19 pandemic. Studies like [8] demonstrated, that Twitter can act as a knowledge translation tool disseminating knowledge to a large group of people. Along with broadcasting COVID-19 information resources, public health can also consider spreading a sense of optimism among people through positive motivating videos, pictures, messages, and more. In addition, public health can continue monitoring people with negative sentiments and deliver targeted information to preserve the mental health of those individuals who show extremely negative sentiments. Further, the number of positive and negative sentiments can help public policymakers assess compliance and non-compliance in practicing preventive measures. For more than fifteen years [9], a large number of scientific works using data from blogs and social media for a multitude of purposes have been published, demonstrating the utility and legitimacy of the use of such data in many fields, especially computer science and linguistics. One of the most popular sub-fields among these, Natural Language Processing (NLP), has suffered a dramatic computational evolution [10], resulting in new applications and techniques such as Sentiment Analysis (SA). This technique, also known as Opinion Mining (OM), utilizes a number of computational tools for extracting, classifying and analyzing subjective information contained in verbal data sources such as text, audio, or video. SA-based workflows have particularly profited from the recent advances in performance and ease of implementation of Machine Learning (ML) algorithms, with most studies involving SA being published at the time containing at least one use of an ML algorithm in any of its stages [11]. According to Hemmatian and Sohrabi [12], an OM workflow can be described by a sequential set of steps with individual obligations and the objective of extracting all existing opinions in a given set of documents. Their description encompasses all processes, ranging from data collection from different social media sources to the performance evaluation of the classified opinions.

Given the advantages in terms of simplicity of collection and ease of processing in contrast with other data types with existing algorithms, textual data is most commonly used for SA applications [13]. Over the past years, literature shows that microblogging social media platforms such as Twitter and Reddit are most commonly used for SA applications, mostly due to the accessibility of the data provided through developer-friendly APIs and wide demographic scope [14,15]. Data acquired from these platforms is often referred to as free of charge, though there are specialized online services which offer to perform data mining from various sources for a fee. Applications of SA using social media data can vary greatly depending on the field, with some authors using it for politics [16], finances [17], hospitality reviews [18], public policy [19], and a wide variety of health-related applications [20] including those focused on the COVID-19 Pandemic [21].

While it is evident that less research on SA is performed on languages other than English, the number of work remains significant [22]. This is especially true for Spanish, where research on SA has also seen significant advances in the past 20 years [23]. This also means that, in a similar fashion as with English, a number of methods for sentiment classification have been successfully applied, ranging from lexicon-based approaches [24,25,26] to more recent applications based on ML algorithms [27]. Some of these lexicon-based approaches such as the one proposed by [28] take into consideration the possible effect that regional variants of the Spanish language might have on estimated sentiment. Even more recently, since the introduction of attention models and specifically the Transformer deep learning architecture [29], NLP research has seen drastic improvements in terms of accuracy and efficiency of processing large text corpora. The popular Bidirectional Encoder Representations from Transformers (BERT) architecture, initially released in English [30], has also been adapted to Spanish in a model similar in dimensions to the original BERT-base architecture pre-trained with a large Spanish corpus from Wikipedia and sources from the OPUS Project [31], and another model pre-trained from a dataset of 500 M tweets in Spanish [32]. While DL methods continue to innovate on increasingly complex models, some other more computationally efficient methods such as Gradient Boosting and its variants, have been especially useful in languages where there is a lack of large pre-trained models [33,34,35].

Some of these approaches, and their analogs in languages other than English, have been successfully applied to scenarios related to the scope of our research: the effects of the pandemic and massive social events on the general public sentiment of a determined territory. In an effort to measure the emotions expressed by residents of 10 Italian cities during the early months of the pandemic, Fernandez et al. performed a lexicon-based sentiment analysis of more than 4 million coronavirus-related tweets [36], with results pointing at strong emotional responses towards health policies in the country. A similar analysis was also carried out by Kydros et al. in Greece during the acute phase of the pandemic [37]. The authors of this work employed a lexicon-based approach on approximately 80 thousand tweets corresponding to the early acute phase of the pandemic, resulting in the observation of an overall sentiment of fear during the period of analysis. In Jordan, Obiedat et al. [38] implemented an evolutionary approach of sentiment analysis of Arabic language during the first half of 2020, that when used to determine the emotional evolution of the country during the period of study, pointed towards a shift in interest from the disease itself to the measures taken by the government to attempt to control the spread of the disease. In China during the early months of the Pandemic, Wang et al. extracted approximately one million microblogging posts from the Chinese social media platform Weibo and used a fine-tuned BERT model to obtain the earliest most discussed topics and estimate their respective sentiment polarities [39]. The authors describe a sharp uptick in negative mentions related to raw meat and bats, probably referring to the first theories of origin of the virus.

A number of recent historical events around the world have presented themselves as opportunities for exploring the use of social media SA as a tool for measuring public sentiment during periods of social unrest. After the Hong Kong Protests of 2019, Qi et al. processed Twitter and Reddit data related to the protests, finding out negative sentiment relationships of varying temporal lengths in topics related to the Hong Kong police force and the government of the territory [40]. To analyze the influence of Twitter as a tool in the context of the 2011 Egyptian revolution, Bang et al. proposed the use of an SA workflow to classify the sentiment and emotions expressed by Egyptian or Egypt-related users during this crisis [41]. The authors described a predominance of anger, fear, pride, and hope emotions among the analyzed tweets both in Arabic and English language. In another recent effort to quantify the relationship between social media activism and real-life protests during the height of the #JusticeForGeorgeFloyd movement in the United States, Nicoletti et al. [42] used a combination of data sources such as spatial and demographic data, geo-tagged Tweets, and physical protest reports from the period of interest. The authors findings suggested that by modeling the relationship between these variables, it is possible to predict protest activity on a selected period of time based on the frequency of social movement related tweet counts from before such a period.

A more recent, massively disruptive event: the January 6th riots of the U.S. Capitol, has also been the subject of SA studies as it is now known that social media played an instrumental role in the communication and incitation of rioters. In a study by Jakubik et al., over 700,000 posts from the Parler social network produced on the day of the riot were analyzed, detailing the temporal progression of basic and derived emotions such as joy, fear, surprise, disapproval, unbelief, outrage, and guilt [43]. On another study by Li et al., a number of multimedia sources including videos, tweets, and speeches, were analyzed by means of Granger causality methods [44]. The authors of this study claim that ex-president Donald Trump’s tweets and speeches could have been used to predict escalations in levels of violence among rioters the day of the event, as there is a relationship between the behavior of figures of authority on social media and the level of violence in real-life events.

In the Republic of Panama, a high income country with significantly unequal distribution of wealth [45], it is estimated that an average of 63% of the population has access to a broadband internet connection [46]. Since the year 2019, this country has witnessed a number of large-scale events which evoked strong public opinions. Some of these were especially polarizing, such as the arrival and departure of over 70 thousand catholic pilgrims through the capital Panama City in less than fifteen days, the national presidential elections, and the outburst of large-scale protests in response to corruption allegations of the newly elected national assembly. These events, in combination with the long-lasting mobility and alcohol consumption restrictions imposed by the Panamanian government in 2020 as measures to contain the spread of the pandemic in the country, most definitely had an impact strong enough to be easily reflected on the public opinion.

The goal of the present work is to extract, quantify, process, and analyze large amounts of Twitter data produced by residents of Panama in Spanish during the years 2019 and 2020, in order to prove if the suspected events of impact did produce an observable response on the general population sentiment, while analyzing the performance of popular ML algorithms in SA workflows when applying them to a regional variant of Spanish text. In order to determine the best possible algorithms for sentiment extraction and classification, a small comparative analysis between popular ML algorithms used for SA is also to be performed, resulting in a second possible contribution for this work. From here on, this paper is structured as follows: The remaining content of Section 1 will provide a brief insight into the state of the art of sentiment analysis applications related to the objectives of this work, Section 2 provides a detailed explanation of the all the methods used for the acquisition and processing of the data of interest, while the results of all these methods as well as the results of the preliminary comparative results are presented on Section 3. The analysis, contributions and conclusions drawn from the presented results are described on Section 4, with Acknowledgments and References immediately following.

## 2. Materials and Methods

As mentioned above, the main goal of this study is to extract and process Twitter data from residents of the Republic of Panama during the years 2019 and 2020. In order to achieve that, a series of sequential tasks comprising extraction, pre-processing and classification are performed, and described in this Section.

### 2.1. Data Acquisition

According to the International Telecommunication Union (ITU) in 2019, Panama’s 2.7 million out of its approximately 4.2 million inhabitants use their connection to the internet [46], amounting to approximately 64% of the population. When sources external to both the government and the social media platforms themselves extrapolated information provided by social media platforms earnings announcements and media reports, it was found that Panama could have approximately 2.82 million active social media users [47]. This number does not necessarily equal individual users, as each user could have multiple social media accounts. The same report states that approximately 406 thousand of these active users are on Twitter, making up a 12% of the national population. This approximate number constitutes our starting point for compiling a list of Panamanian Twitter users from which tweets will be extracted.

First, a list of the 25 most followed Panamanian Twitter accounts was compiled, and from those it was found that 10 satisfactorily comply with a series of established criteria. These criteria aimed to consider accounts with lack of a strong influence abroad that could imply large numbers of foreign followers, and accounts with no obvious political affiliations or motivations that could possess large numbers of automated and/or inactive followers. These criteria thus excluded famous Panamanian artists, politicians, and government accounts with political reasons to non-transparently inflate their follower counts. Once the list of Panamanian influential accounts was compiled, a third party web scraping service was used to extract the complete follower list of the selected influential accounts. These follower lists were then cross-referenced and usernames that appeared in more than three of the follower lists were stored in a separate list, as these were very possibly Panamanians or residents of Panama. The finished list of Panamanian Twitter users consisted of more than 1.5 million accounts.

With the finished user list, a user-centered Tweet extraction could be performed. For this extraction, the Twitter API v2 [48] with an Academic Research access level was used. This level of access provides the use of the Full-archive search endpoint, which allows the extraction of tweets from any point in time since the first tweet ever published, limited only by the number of requests per 15-min window and a monthly tweet cap of 10 million tweets. Once the access was obtained, we proceeded to extract all the tweets produced by the first 400 thousand twitter users from our list, as they’re more recent and less possibly inactive accounts, on a yearly basis between 2019 and 2020. The user-based extraction was carried out using a script written in the Python programming language, whose logic is described in Algorithm 1:
**Algorithm 1** Tweet Extraction Algorithm1:tweets = id, text, hashtags, date, geo, like, quote, reply, retweet2:savedtweets = id, text, hashtags, date, geo, like, quote, reply, retweet3:Read userlist4:**for** $userlist=user_{1},user_{2},...,user_{n}$ **do**5:      **if** $user_{n}$ has tweets **then**6:           Extract tweets from $user_{n}$7:           Append tweets to savedtweets8:           Clear tweets9:    Sleep for 3 s

**Figure 1:**
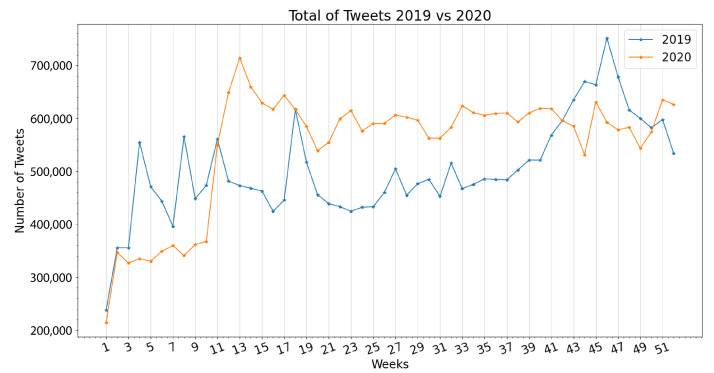
Weekly distribution of extracted tweets.

In this algorithm, *tweets* is a vector that temporarily stores each user extraction with each of the extracted fields (tweet ID, text of the tweet, hashtags used, date of publication, geographical information, likes, quote retweets, replies, and retweets), and *savedtweets* is a vector that permanently stores every extraction. All the information found in the aforementioned fields of every tweet is stored, but not all of it is to be immediately used. To comply with the time rate limit imposed by Twitter, the algorithm waits for 3 s after each extraction, allowing it to run uninterrupted for weeks until completion of the monthly tweet cap, and subsequently until the end of the selected portion of the user list. After months of repeating this process, the complete list of tweets from the selected authors had produced over 26 million tweets from 2019 and over 28 million tweets from 2020. Figure 1 shows the weekly distribution of the tweets extracted by year, with the first week of both years displaying a drastically smaller number of tweets as a result of a smaller number of calendar days during these weeks.

### 2.2. Data Pre-Processing

**Figure 2:**
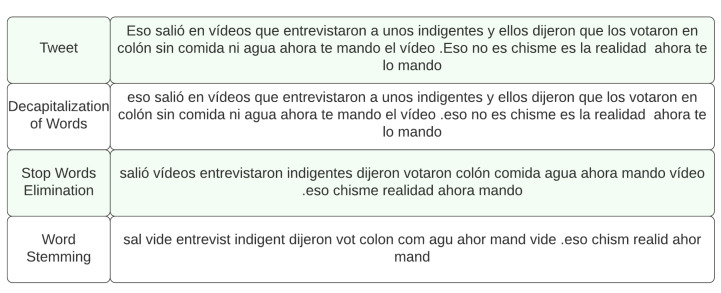
Example of the stages of tweet pre-processing.

Once the data has been correctly extracted and quantified, it’s necessary to adequately condition it for further processing through a series of steps. The objective of this stage is to maximize the performance of the processing algorithms in further stages, and to ensure that the data is representative enough of the population in question. After the text field of the stored tweets has been extracted, duplicates and retweets are deleted, and the textual data is cleaned by removing links, de-capitalizing words and replacing newline characters with spaces. Once the text has been cleaned, stop words are removed using a list of common Spanish stop words, and the remaining words are stemmed down to their roots in an effort to reduce the dimensionality of the dataset. Finally, the LangID [49] language identification tool is used to eliminate tweets that may not be in the Spanish language, since they would not contribute to this specific analysis. In the cases where any tweets were left without textual data after these processes, their entries were deleted from the analysis dataset as well. Figure 2 shows an example of the data cleanup performed on a tweet after each consecutive step.

After successfully cleaning and filtering the extracted text, word vectors were created using the Count Vectorizer tool from the Scikit-Learn [50] Python library. This tool converted the pre-processed text of each tweet into an array of tokens, easing and normalizing the process of training any algorithm used for SA.

#### 2.2.1. Immigrant Bias

For centuries, the Republic of Panama has been an important logistical hub with particularly large waves of immigration in different periods of its history due to its geographical position and the services provided by the Panama Canal. More recently, and as a consequence of the Venezuelan migration crisis [51], Panama has received a large number of Venezuelan migrants, with a UNICEF estimate placing the number of migrants at almost 100 thousand by the end of 2018 [52]. As it has been observed in other parts of the world that have also experienced significant migrant influxes [53,54], migrants and refugees are avid social media users, as it allows them to receive information from their countries of origin and relatives based on existing social ties. According to our preliminary findings, Panama appears to not differ from the observations of these studies.

An analysis of the most frequently mentioned words in tweets extracted from the fourth week of 2019, revealed the discrepancy of opinion between Panamanians and Venezuelans living in the country at the time. During that week, two different massive events were taking place in both Venezuela and Panama. In Panama, the World Youth Day 2019, an event perceived as positive by Panamanians, saw the arrival of Pope Francis accompanied by more than 70 thousand catholic pilgrims from 150 countries. In contrast, during the same period of time, the Venezuelan presidential crisis had just begun; an ongoing political crisis generally perceived as negative by Venezuelans. In an attempt to maintain the sentiment analysis of tweets on topics related to the Republic of Panama, we decided to filter tweets containing words related to the Venezuelan presidential crisis, such as “Maduro” (referring to Nicolás Maduro), “Guaido” (referring to Juan Guaidó), “Ayuda Humanitaria” (referring to the humanitarian aid sent notoriously by the United States and its allies during the start of said crisis), and other words frequently associated with this topic.

**Figure 3:**
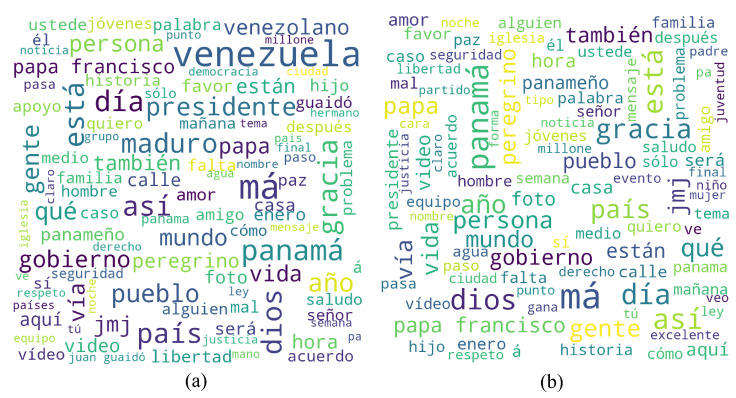
Word clouds made from most frequent words used during the fourth week of 2019 both (**a**) before applying a immigrant bias filter and (**b**) after applying a immigrant bias filter.

Figure 3 shows word clouds made from the most frequently mentioned words in tweets during the fourth week of 2019, before and after filtering for the immigrant bias. Before applying said filter, words such as “venezuela”, “maduro”, and “presidente” appear notoriously frequently, while after filtering, words such as “peregrino” (pilgrim), “jmj” (JMJ or World Youth Day), “panamá”, and “dios” (god), appear to be the most frequently mentioned.

### 2.3. Sentiment Analysis

**Figure 4:**
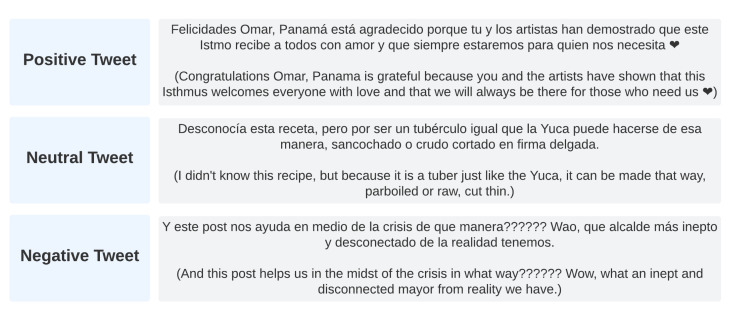
Examples of tweets classified as positive, neutral, and negative respectively.

For the classification of sentiment from vectorized tweets, we propose the application of one of six ML algorithms commonly used in SA workflows. To train these algorithms, an extract of 8000 tweets from the large tweet dataset was manually labeled and independently reviewed by three human reviewers. These tweets were labeled as either exerting a negative, neutral or positive sentiment. From the 8000 extracted tweets, 3314 were assigned the same label by all three human reviewers, leading us to the conclusion that this was the most possible sentiment reflected by each individual tweet. An example of tweets from each categories is shown on Figure 4. Once labeled, the manually reviewed dataset consisted of 1906 tweets found to have a negative sentiment, 466 tweets found to have a neutral sentiment, and 942 tweets found to have a positive sentiment.

As mentioned, six popular ML algorithms were selected for a small comparative analysis of their respective performance when classifying the sentiment of the flagged tweets. The selected algorithms were: Random Forest, K-Nearest Neighbors, Naive Bayes, Gradient Boosting, Stochastic Gradient Descent, a Support Vector Classifier, and Extreme Gradient Boosting (XGBoost). The hyperparameters of each of these models (with the exception of Naive Bayes) were obtained by executing grid searches using an 80%/20% train/test partition, until reaching the combination which could result in the best possible performance. The optimal hyperparameters for each of the selected algorithms are presented below.

#### 2.3.1. Random Forest

A random forest (RF) is an algorithm that can be used for both classification and regression problems. It operates by constructing a set of decision trees, where each tree gives a prediction for a given tweet using features chosen at random from the total set of features available. The final model is then created by averaging the predictions from all of the individual trees to create a more robust prediction. Random Forests present the advantage of being significantly less prone to overfitting than other machine learning algorithms, improving model generalization capabilities. Following the grid search operation, the optimal parameters that resulted in the highest accuracy on the training set of tweets include the ’gini’ criterion to measure split quality, the maximum depth of the tree was set to 10, the maximum features to consider for a split was set to ’auto’, and the number of trees in the forest was set to 10.

#### 2.3.2. K-Nearest Neighbors (KNN)

KNN is an NLP method for classification or regression problems that uses a vector space model to represent tweets as points in a high-dimensional space. In this representation, the smallest distances between a given tweet of unknown sentiment and tweets with labeled sentiment data are then used to determine the sentiment by assigning the unknown tweet the majority sentiment from its K nearest neighbors. The grid search operation yielded the best results when using the Euclidean distance metric, weighing the labeled tweets by their distance to the tweet to be classified, and considering the 7 nearest neighbors.

#### 2.3.3. Naive Bayes

The Naive Bayes method aims to determine the probability of a particular event occurring, based on the occurrence of other events. In the context of the current work, this can be used to calculate the likelihood that a given text document expresses positive or negative sentiment, based on the presence of certain words or phrases. To do this, the Naive Bayes algorithm first builds a model by calculating the probabilities of various events occurring within a set of training data consisting of tweets that have been labeled as having a positive or negative sentiment. Once the model has been built, it can then be applied to new text documents in order to predict their sentiment.

#### 2.3.4. Gradient Boosting (GB)

Gradient Boosting is a machine learning algorithm which is often used to create predictive models. GB operates by sequentially adding decision trees to the final model, where each tree attempts to correct errors made by its predecessor. For example, if the first tree in a sequence classifies negative tweets as positive or vice versa, this will be corrected in subsequent iterations of the algorithm using new data sets obtained from Wikipedia that have been labeled manually with sentiment scores. To define what an error actually is and thus build the decision trees for boosting, GB uses differentiable loss functions such as adaptive gradient descent. It was selected as one of our methods due to its accuracy relative to computational requirements when compared to other machine learning algorithms when predicting text document sentiment. The optimal parameter combination resulting from a grid search operation were the Mean Square Error with Friedman improvement score criterion, a 0.15 learning rate, maximum depth of 8, maximum features considered were the square root of the total feature count, 200 estimators. Finally, 80% of samples were used to fit individual base learners.

#### 2.3.5. Stochastic Gradient Descent (SGD)

SGD is another method that may be used to solve both classification and regression problems [55]. Following the definition of an objective function, the SGD algorithms iteratively updates parameters while moving towards some global optimum point on said function. By randomly selecting training examples from a set of labeled tweets and then updating the current model, the SGD algorithms converges to a solution that better fits these examples. This process continues until some stopping criterion is met, in other words, the set of parameters gradually converges through iterations towards values which correspond to the global minimum for the selected objective function. To do this, a learning rate parameter is used which controls how fast or slow these updates happen [56]. The best performing model according to the grid search operation used a logarithmic regression loss function, had a maximum number of iterations set at 5, and used an L2 norm penalty.

#### 2.3.6. Support Vector Classifier (SVC)

The SVC algorithm operates by constructing a decision boundary in the shape of a hyperplane, which separates two regions of points in a vector space of dimensionality equal or higher than that of the vectorized tweets. By defining these hyperplanes such that the margins between them are maximized, the SVC algorithm aims to create a model which is robust against small variations in input data. The best performing model was a Support Vector Classifier with a C regularization parameter value of 10, a gamma value of 0.1, and an RBF kernel.

#### 2.3.7. Extreme Gradient Boosting (XGBoost)

XGBoost is a machine learning algorithm that operates by building a series of decision trees where the output of each tree depends on the output of the previous one. It does this by sequentially learning from previous mistakes, which it does by iteratively calculating a gradient of a given loss function, which is used to update the model in order to reduce the gradient until some stopping criteria is met. An important advantage of the XGBoost structure when compared to RFs or SVCs is its ability to parallelize computations across multiple cores which decreases the total training time for a given training set. The fraction of columns to be randomly sampled for each tree was set to 0.5, the learning rate used was 0.05, the maximum depth of a tree was set to 10, the number of trees in the forest was set to 1000 and the subsample ratio of rows per tree was set to 0.9. The objective function used was a softmax multi-class objective function and the number of classes in the dataset was 3. The mean error metric was used to evaluate performance.

With all the grid searches performed, the best performing combination of hyperparameters of every algorithm was then tested on a smaller evaluation dataset of 300 tweets (100 negative, 100 neutral, and 100 positive), which was also labeled by three different human reviewers, and which is not part of the larger train/test dataset. This evaluation was performed on a desktop computer equipped with an 11th Gen. Intel Core i7 processor, 16 GB of installed DDR4 RAM, and an NVIDIA GeForce RTX 3060 Ti graphics card.

Using the best performing algorithm with the best combination of hyperparameters obtained from the grid search, a sentiment index estimation was performed on the large unlabeled tweet dataset, with a weekly granularity. Such a sentiment index *SI* is defined as the difference between the number of tweets categorized by the algorithm as negative minus those categorized as positive, divided by the total of tweets used during the analysis window including neutrally-classified tweets, also shown in Equation (1).

(1) $$SI=\frac{negative\;tweets-positive\;tweets}{total\;of\;tweets}$$

Once the weekly *SI* is estimated for all 104 weeks spanning the years of the analysis, it is possible to compare abrupt changes in sentiment with events in the Republic of Panama that may have influenced the generalized sentiment of the users considered for the study.

## 3. Results

In this paper, we implemented an SA-based workflow for the analysis of Spanish-language tweets from the Republic of Panama. This workflow was tested by analyzing tweets from 2019 and 2020, two years which saw a high number of disruptive events happening in the country and abroad. This section describes the most relevant findings of such analysis in their respective stages.

### 3.1. Extracted Data

**Figure 5:**
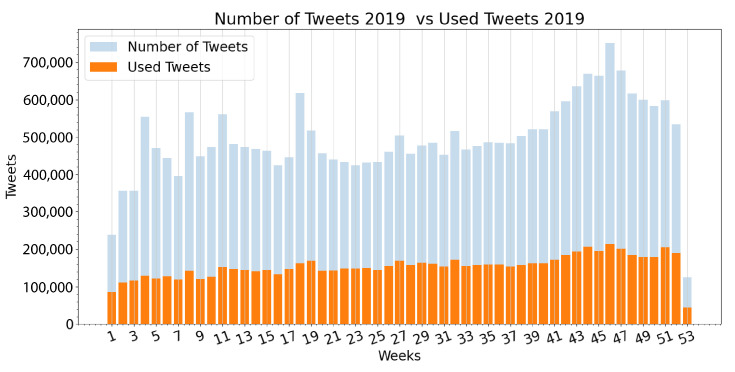
Number of tweets extracted versus number of tweets used in 2019.

**Figure 6:**
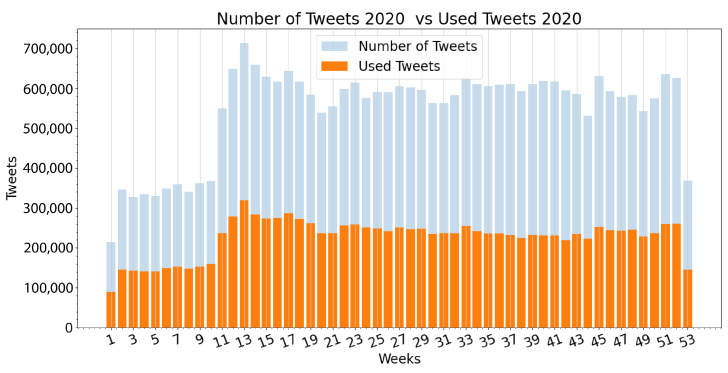
Number of tweets extracted versus number of tweets used in 2020.

As stated in Section 2, a robust pre-processing stage is implemented immediately after extraction. This resulted in a large number of tweets not being used for the analysis because of their content, or lack of. In 2019 for example, 26,280,472 tweets were extracted while only 8,157,634 were used for the analysis. For 2020, the number of extracted tweets was 28,921,939, while only 12,010,461 ended up being used. In order to visualize how much data was used for the analysis when compared to the amount of data originally extracted, Figure 5 and Figure 6 compare these exact quantities on a weekly basis.

### 3.2. Method Evaluation

After performing the hyperparameter grid search on all ML algorithms, it became evident that most of the evaluated methods performed satisfactorily. For this comparison, evaluation accuracy, precision, recall and F1 scores are obtained, as well as the time it took each algorithm to compute all 300 samples in the evaluation dataset. As shown in Table 1, the XGBoost algorithm outperformed the others in evaluation accuracy, precision, recall, and F1 score, while being third in inference time to the KNN and SGD algorithms.

**Figure 7:**
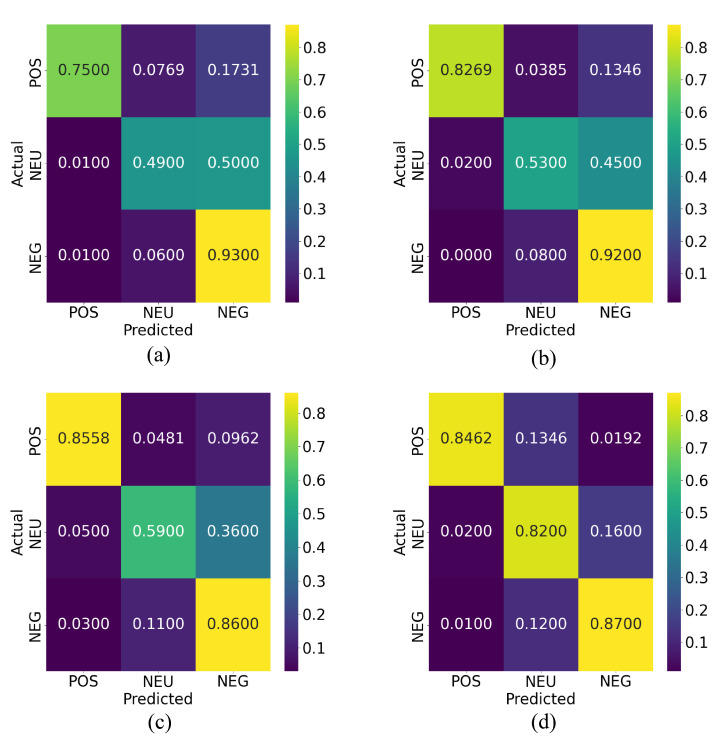
Confusion matrix obtained from the evaluation of the (**a**) Gradient Boosting algorithm, (**b**) Stochastic Gradient Descent algorithm, (**c**) Support Vector Classifier algorithm and (**d**) XGBoost algorithm.

Confusion matrices for the four best-performing algorithms in the analysis were subsequently generated by comparing the predicted labels with the ground truth provided by the evaluation dataset. These are displayed in Figure 7. By comparing such confusion matrices, it was evident that all but the XGBoost algorithm performed poorly when classifying tweets flagged as neutral. This behavior is expected, and concurs with a similar pattern observed among human reviewers when flagging the training and evaluation dataset, where neutral tweets were the least agreed-upon category among all reviewers. Given these results, it was determined that the XGBoost algorithm would be used for performing the sentiment index estimation for the large dataset.

### 3.3. Sentiment Index Estimation

**Figure 8:**
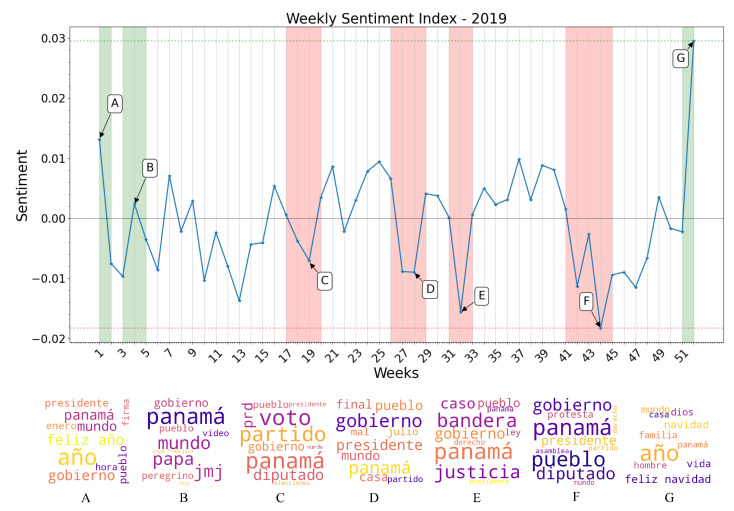
Sentiment index values and word clouds generated from most frequent words used each week, 2019.

**Figure 9:**
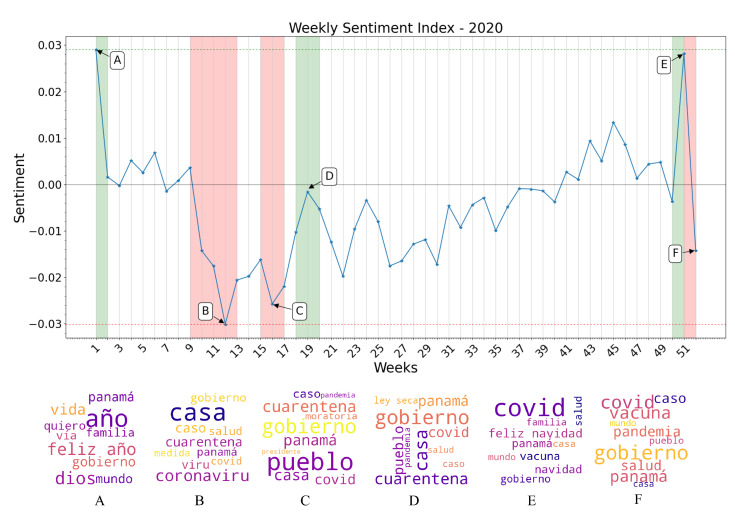
Sentiment index values and word clouds generated from most frequent words used each week, 2020.

After determining that the XGBoost algorithm would be used to classify the sentiment of tweets contained in the large dataset, a sentiment index value was estimated for all the weeks of interest. As it is shown in Figure 8 and Figure 9, the results of this classification were plotted with a weekly granularity for the years 2019 and 2020. When observing each curve individually, a number of strong sentiment inflections can be pointed out on certain weeks for each year. To trace the cause of these inflections, the most frequently appearing words in each week are obtained, and newspapers and digital outlets are compared with these words to find a possible correlation between sentiment inflections and events taking place in the country. For the year 2019, the most possible causes of certain sentiment inflections found after this analysis are listed and described as follows:

- Week 1: New Year celebrations and discussions of yearly resolutions predominate the public discussion, raising the overall sentiment towards a positive polarity. Notice the words “feliz año” (“happy new year” in Spanish) in the word cloud generated from the specified time period.
- Week 4: World Youth Day (WYD) 2019 takes place in Panama City from the 22 to the 27 of January. Pope Francis arrives on the country on the 23rd of January as well as more than 70 thousand foreign pilgrims. Sentiment regarding the event is overall conflicting, but tends towards a positive polarity. Notice the words “papa francisco”, “jmj” and “peregrino” (“Pope Francis”, “WYD”, and “pilgrim” in Spanish) in the word cloud of this period.
- Weeks 18–19: General elections take place in Panama. President Laurentino Cortizo is elected with 33% of votes in his favor, and his party (PRD) also obtaining a majority of legislators in the National Assembly, causing an overall discontent among the majority of the population with an online presence. Notice the words “diputado”, “presidente”, “prd”, “voto”, “party” (“depute”, “president”, “vote” and “[political] party” in Spanish) in the word cloud generated.
- Weeks 27–28: The President Elect, and newly elected National Assembly assume office. Public discontent grows, as a viral campaign against re-election of legislators fails. Notice the words “gobierno”, “presidente”, “pueblo”, “mal” (“governement”, “president”, “people”, and “bad” in Spanish) in the generated word cloud.
- Week 32: Protests against the re-election of legislator Benicio Robinson and his fellow party members take place outside the National Assembly. During these protests, an activist group displayed an altered version of the national flag with the LGBT colors instead of the red and blue, causing discontent among a mostly conservative population. Notice the words “bandera”, “justicia”, “gobierno”, and “pueblo” (“flag”, “justice”, “government” and “people” in Spanish) in the generated word cloud.
- Weeks 41–44: Mass-scale protests against the elected National Assembly erupt, resulting in dozens of arrests on consecutive days, and the deployment of riot control police on multiple locations. Most negative polarity of the year. Notice the words “protesta”, “gobierno”, “pueblo”, “diputado” and “presidente” (“protest”, “government”, “people”, “depute” and “president” in Spanish) in the word clouds generated.
- Week 52: Christmas and New Year celebrations raise overall sentiment once again. Notice the words “feliz navidad” (“Merry Christmas” in Spanish) as some of the most mentioned during this week in the word clouds generated.

For the year 2020, the most possible causes of certain sentiment inflections found after this analysis are listed and described as follows:

- Week 1: Year-end holiday sentiment carries on to the first week of the year. Notice the words “feliz año”, “quiero” and “familia” (“Happy New Year”, “I want”, and “family” in Spanish) as the most mentioned in this time period reflected in the word cloud.
- Week 10–13: First official detection of a COVID-19 case in Panama, and declaration of a Pandemic by the WHO. Panama’s Ministry of Health begins holding daily press conferences on the worsening epidemic situation in the country. Citizens demand the government to dictate quarantine measures to curb the spread of the disease (Week 12). Most negative sentiment polarity of the year. First measure of quarantine and curfew on a national level, as well as massive business closures and a prohibition of alcohol sales (Week 13). Sentiment remains negative, but less than the previous week as the government enacted restrictive measures to curb the spread of the disease. Most mentioned words according to the word clouds are “cuarentena”, “coronavirus”, “caso”, and “casa” (“quarantine”, “coronavirus”, “case” and “home” in Spanish) the last one referring to the “stay at home” movement seen worldwide during the first months of the pandemic.
- Week 16: Quarantine and curfew measures are extended beyond their original estimates. As the work situation of the majority of Panamanians remains uncertain, demand grows on social media for government-enforced moratoriums and economic aid. Sentiment remains negative. Among the most mentioned words remain “cuarentena”, “casa”, “covid”, “caso”, and “gobierno” as well as “moratoria” and “pueblo” (“quarantine”, “home”, “covid”, “case”, “government”, “moratorium” and “people” in Spanish).
- Week 19: Prohibition of alcohol sales is lifted. Sentiment tends toward a more positive polarity, criticism towards the government remains. Most frequent words remain stamble in comparison with previous weeks, with an uptick in metions of “ley seca” or “dry spell” in Spanish, meaning prohibition of alcohol sales.
- Week 51: The prospect of Christmas celebrations raise overall sentiment. Government confirms the emergency approval of the Pfizer-BioNTech vaccine, weeks after confirming the initial acquisition of 3 million of their vaccines, also contributing to an uptick in positive sentiment. Words such as “feliz navidad” and “vacuna” (“merry christmas” and “vaccine” in Spanish) appear frequently.
- Week 52: New wave of cases causes the installment of two-week quarantine and curfew measures on a national level in an attempt to control rising case numbers. The Panamanian government publishes the initial schedule of vaccine distribution, causing debate on social media. Sentiment deteriorates once again towards a negative polarity. Some of the most frequent words used according to the word clouds generated are “covid”, “vacuna”, “pandemia” and “caso” (“covid”, “vaccine”, “pandemic” and “case” in Spanish), not deviating from earlier stages of the pandemic.

#### Impact of Immigrant Bias

**Figure 10:**
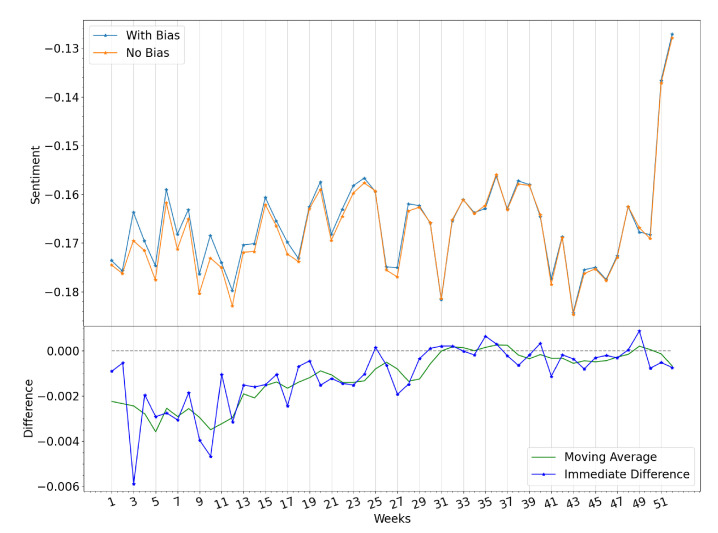
Sentiment index in 2019, with and without accounting for the immigrant bias, as well as the immediate difference in sentiment.

As mentioned in Section 2.2.1, an immigrant bias is thought to be always observable in social media data extracted from countries with large immigration events, as it is the case with Panama. Within the scope of the weeks included for the analysis, and as a result of the Venezuelan Presidential Crisis, a divergence in sentiment between suspected Venezuelans and Panamanians is exhibited especially during the first 27 weeks of the year 2019, as it is displayed in Figure 10. Upon analysis of events in both countries, possible causes of these divergence effects have been found, and will be mentioned as follows:

- Week 3: Juan Guaidó is sworn Acting President of Venezuela on the 23rd of January, 2019. Millions of Venezuelans participated in massive demonstrations in Venezuela and in various countries around the world.
- Week 10: Nationwide blackouts occur between the 7th and the 12th of March resulting in at least 43 deaths related to a lack of power for critical equipment in venezuelan hospitals. This was the largest power outage in the country’s history, and even affected neighboring regions of Brazil.

## 4. Discussion of Results

The extraction and classification of sentiment from text has greatly improved as a result of the development and implementation of ML algorithms into NLP applications. Even when taking into account the different performance results obtained when comparing specific algorithms, it’s evident that most of them are capable of accurately distinguishing between the polarity of sentiments, at relatively low inference times. However, as the results displayed on Section 3.2 show, the XGBoost algorithm was able to achieve higher neutral-sentiment classification accuracy when compared to the other explored algorithms. This behavior was to some extent, expected, as the XGBoost algorithm is an ensemble method which performs particularly well on data with large numbers of observations given its capacity to use many trees to make a decision in a time-efficient manner. The experiments performed confirm that in this specific case of study, these features of the XGBoost algorithm allowed it to adapt to the data better than other highly popular but less optimized algorithms such as the SVM-based classifier or the non-extreme version of the Gradient Boosting algorithm. When analyzing the specific case of the SVM classifier, for it to reach its maximum accuracy it becomes necessary to project to a high-dimensional space for it to be able to linearly separate the data, compromising algorithm complexity without a substantial benefit in accuracy. Nonetheless, we consider it to be good starting point in any exploratory analysis as generic SVM kernels available in programming interfaces have proven to be good at adapting to many different kinds of data. We also consider the results obtained from the SGD-trained logarithmic regression algorithm to be of particular interest, as the algorithm correctly learned to distinguish between negative and positive polarities with an inference time much lower than all the other algorithms studied. This is probably product of the low computational complexity of a logistic regression algorithm combined with the optimized learning of the SGD algorithm. Having implemented the proposed workflow, we have proved that it is possible to estimate a specific population’s sentiment polarity based algorithms such as the ones compared.

## 5. Conclusions

The large number of tweets obtained through the years 2019 and 2020 on itself can be sufficient proof that the decision to extract tweets on an author-centered basis is effective and representative of the population of Panama. This is evident when analyzing weekly tweet frequencies during specific events of interest, such as the imposition of the first quarantine measures in the country on March of 2020, or the large-scale protests on November of 2019. Word clouds made from the extracted tweets on a weekly basis also shed light on the strong influence that massive immigration can have on the national opinion of a country. In some cases, particular events on the countries of origin of the immigrant population could significantly alter the sentiment index of a given week. These events can have particularly marked effects in countries such as Panama that have relatively small populations and average levels of access to the internet.

We believe that a workflow such as the one presented carries an inherent importance for non-English speaking governments or organizations from smaller countries where geo-filtering tweets or other textual data is not feasible. This presents a particular potential in the Latin American region, where local Spanish jargon can vary considerably from country to country. The decision to extract tweets through a user-oriented and not keyword or geotag-based algorithm proved to be an effective method to capture the widest possible variety of topics and highest quantity of tweets. The implementation of open population-level sentiment monitoring could be beneficial for critical decision making on a government level, as it would allow anonymous generalized feedback on policy changes or events that affect entire countries. Regarding future developments of the presented work, the authors consider that this workflow can be further improved by implementing changes such as the implementation of Deep Learning algorithms for sentiment classification, and possible health-related applications of the finished product. In addition, the exploration of the influence on a variety of independent and inter-dependent demographic variables on observed sentiment, such as age, gender, and socioeconomic status could provide further valuable insights on population sentiment as this data becomes available.

## Figures and Tables

**Table 1:** Preliminary performance comparison between popular classification ML algorithms used for SA, with the most favorable values for each column shown in bold.

Method	Accuracy	Precision	Recall	F1	Time (ms)
**KNN**	0.4703	0.7943	0.4728	0.4106	10
**NB**	0.6282	0.6449	0.6271	0.6189	301
**GB**	0.7401	0.7944	0.7401	0.7373	1894
**SGD**	0.7631	0.8041	0.7620	0.7594	**6**
**SVC**	0.7697	0.7852	0.7685	0.7670	833
**XGB**	**0.8466**	**0.8536**	**0.8466**	**0.8484**	34
